# Inverted pyramid 3-axis silicon Hall-effect magnetic sensor with offset cancellation

**DOI:** 10.1038/s41378-025-00876-9

**Published:** 2025-02-14

**Authors:** Jacopo Ruggeri, Udo Ausserlechner, Helmut Köck, Karen M. Dowling

**Affiliations:** 1 Department of Microelectronics, Delft University of Technology, 2628 CD Delft, The Netherlands; 2 Infineon Technologies AG, 9500 Villach, Austria

**Keywords:** Electrical and electronic engineering, Physics

## Abstract

Microelectronic magnetic sensors are essential in diverse applications, including automotive, industrial, and consumer electronics. Hall-effect devices hold the largest share of the magnetic sensor market, and they are particularly valued for their reliability, low cost and CMOS compatibility. This paper introduces a novel 3-axis Hall-effect sensor element based on an inverted pyramid structure, realized by leveraging MEMS micromachining and CMOS processing. The devices are manufactured by etching the pyramid openings with TMAH and implanting the sloped walls with n-dopants to define the active area. Through the use of various bias-sense detection modes, the device is able to detect both in-plane and out-of-plane magnetic fields within a single compact structure. In addition, the offset can be significantly reduced by one to three orders of magnitude by employing the current-spinning method. The device presented in this work demonstrated high in-plane and out-of-plane current- and voltage-related sensitivities ranging between 64.1 to 198 V A^−1^ T^−^^1^ and 14.8 to 21.4 mV V^−1^ T^−1^, with crosstalk below 4.7%. The sensor exhibits a thermal noise floor which corresponds to approximately $0.5\,\mu \text{T}/\sqrt{\text{Hz}}$ at 1.31 V supply. This novel Hall-effect sensor represents a promising and simpler alternative to existing state-of-the-art 3-axis magnetic sensors, offering a viable solution for precise and reliable magnetic field sensing in various applications such as position feedback and power monitoring.

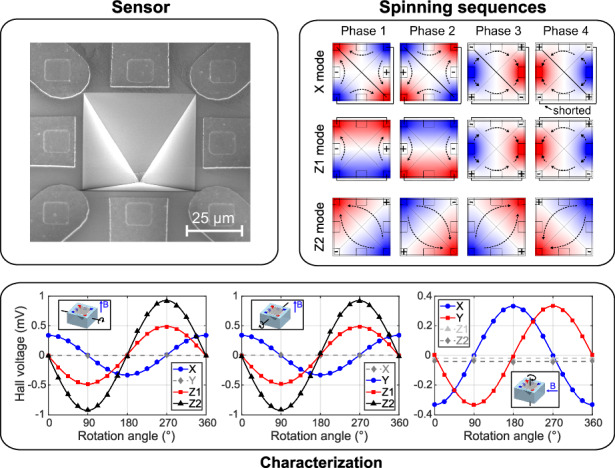

## Introduction

Magnetic sensors serve as fundamental components in a wide array of applications, from industrial and automotive systems to consumer electronics and medical devices. The ability to accurately sense 3D magnetic fields is increasingly crucial, enabling precise position sensing, enhanced motion tracking for robotic and commercial products, contactless angle measurement in automotive steering systems and biomagnetic measurements^1–3^. As technological advancements drive the demand for miniaturization and integration in electronic devices, microscopic and integrated magnetic sensors have become particularly important. The most prevalent types of microelectronic magnetic sensors are magnetoresistive (xMR), micro-electromechanical systems (MEMS) and Hall-effect sensors^4^. Despite the recent progress in MEMS and xMR devices, Hall-effect sensors still hold the largest share of the magnetic sensor market thanks to their durability, reliability, and cost-effectiveness. Compared to their counterparts, Hall-effect devices display lower sensitivity and signal-to-noise ratio, but they are very easily integrated in a complementary metal-oxide semiconductor (CMOS) process, making them a viable and inexpensive solution suitable for commercial applications^5^.

**Fig. 1:**
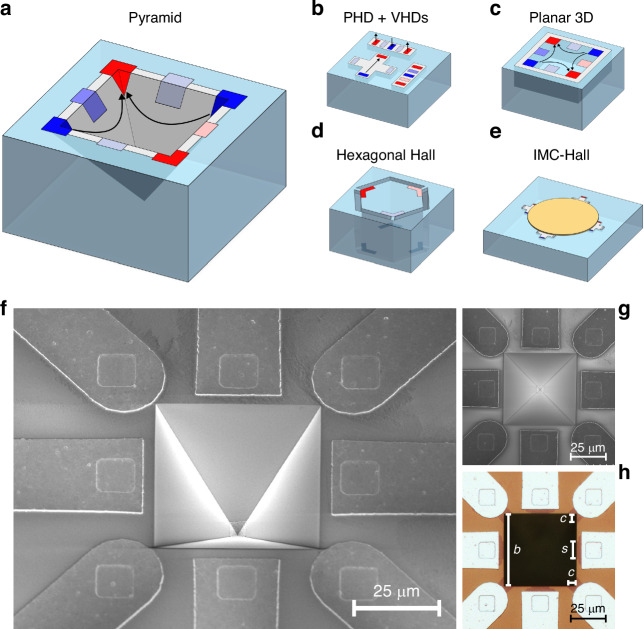
State-of-the-art and 3-axis pyramid device. **a** Our novel pyramid Hall device. **b** A standard 3-axis Hall sensor, composed of one planar Hall device and two vertical Hall devices. **c** The flat 8-contact Hall device, proposed by Schott^9^. **d** Sander’s hexagonal prism Hall device^11^. **e** An IMC Hall device^12^. **f** SEM image of the pyramid device, with a tilting angle of 30°. **g** SEM image of the pyramid device, with no tilting angle. **h** Optical microscope image of the sensor. The pyramid size and the contact dimensions are highlighted in the picture

The two main types of Hall-effect sensors are vertical Hall devices (VHD) and planar Hall devices (PHD). PHDs detect the out-of-plane component of the field, while VHDs detect the in-plane components of the field. When integrated on the same substrate (Fig. 1b) they provide a cheap and simple solution for 3D magnetic field sensing^6^. However, the performance of this configuration is limited by the VHDs. In fact, VHDs require the supply current to flow deep into the active area to reach reasonable sensitivities^7^. This is achieved by means of deep, low-doped wells that, however, are not available in many CMOS technologies. This issue can be solved by scaling down the tub dimensions of the vertical element, but the very close spacing of the contacts causes the device to operate in velocity saturation, which increases the non-ideal effects such as residual offset and flicker noise^8^.

To overcome these issues, as well as to reduce the number of terminals and to reduce footprint, other concepts of 3-axis Hall-effect devices have been proposed. For instance, Schott et al. ^9^ proposed a planar, eight-contact 3-axis Hall-effect sensor (Fig. 1c). This device combines a bottomless Hall plate with coupled 3-contact VHDs to be compact and have high sensitivity. However, similarly to VHDs, it requires deep active areas to operate well. In addition, no current-spinning technique is described, so the low offset and low noise of this device are strictly related to the adopted buried technology, which further limits its integration into a CMOS process. Sander et al. ^10,11^ designed and manufactured a six-contact hexagonal Hall device (Fig. 1d), with high sensitivity and isotropy. In addition, the device can be current-spun to modulate offset and flicker noise. However, the device has a large footprint and the fabrication is quite complex since it involves double-sided patterning, implantation, and deep reactive ion etching.

Lastly, Popovíc’s approach to the problem was to deposit, in a post-processing step, a ferromagnetic material called integrated magneto-concentrator (IMC) on top of several Hall plates^12^. An example of IMC sensor is represented in Fig. 1e. The ferromagnetic material converts locally the in-plane magnetic field into an out-of-plane magnetic field, which can be detected by combining the output of several PHDs. This configuration presents the high sensitivity and low offset typical of Hall plates, and it can be integrated in a CMOS process with few post-processing steps. However, these devices may exhibit hysteresis issues, higher temperature drifts, and saturation at high magnetic fields due to the use of ferromagnetic materials. In addition, this sensor is inherently anisotropic^13^. In this work, we present a novel type of 3-axis Hall-effect sensor, based on an inverted pyramid structure. Simple MEMS micromachining with anisotropic etchants, also adopted in other 3-axis magnetic sensing technologies such as xMR^14^, is employed to manufacture the device represented in Fig. 1a. This sensor can detect the three components of the magnetic field within a single, compact structure using different biasing and sensing configurations, also called modes. Previously, we demonstrated the basic device concept by employing three different modes to perform simple magnetic field measurements^15^. Now, we additionally prove that each of these modes can be current-spun, which means that the offset can be reduced by one to three orders of magnitude. This is a notable result, since offset calibration is a time consuming, costly process in industry which additionally requires zero background magnetic field in the production line. Current-spinning removes this need, improving production efficiency. In addition, we characterize multiple first-generation devices to extract the current-spun sensitivity and residual offset, as well as the crosstalk and the noise power spectrum.

## Sensor concept

### Sensor microstructure

Scanning electron microscope (SEM) and optical microscope images of the sensor are displayed in Fig. 1f–h. The manufactured and characterized device is an eight-contact inverted pyramid with a characteristic angle of 54.74° and a square base *b* of 50 μm. Four contacts are positioned at the corner of the base, while the remaining four are located at the midpoint of each side. The active area is defined by doping the sloped sidewalls, resulting in a pyramid-shell structure. The contact locations and dimensions are defined by n+ implantations, which partially overlap with the active area to ensure electrical connectivity and ohmic contacts. The side wall contacts (*s*) are 10 μm wide and the corner contacts (*c*) are 5 μm. These heavily doped regions extend outside the active area to facilitate the connection with the metal traces, avoiding patterning issues related to the sloped walls. The entire device is covered by a passivation layer of silicon oxide. The contact between the metal traces and the n+ regions is performed through contact openings or vias in the passivation layer. The metal traces extend away from the pyramid structure and terminate with contact pads for further packaging.

### Working principle

To clearly explain the working principle of the device, let us consider an approximate model with three key assumptions:

1. The four sloped faces of the inverted pyramid are separate Hall elements, each slanted at an angle of 54.74°.
2. The four devices are identical, and no mismatch due to processing is present.
3. Each device is ideal with no doping inhomogeneities or lithography inaccuracies/errors.

**Fig. 2:**
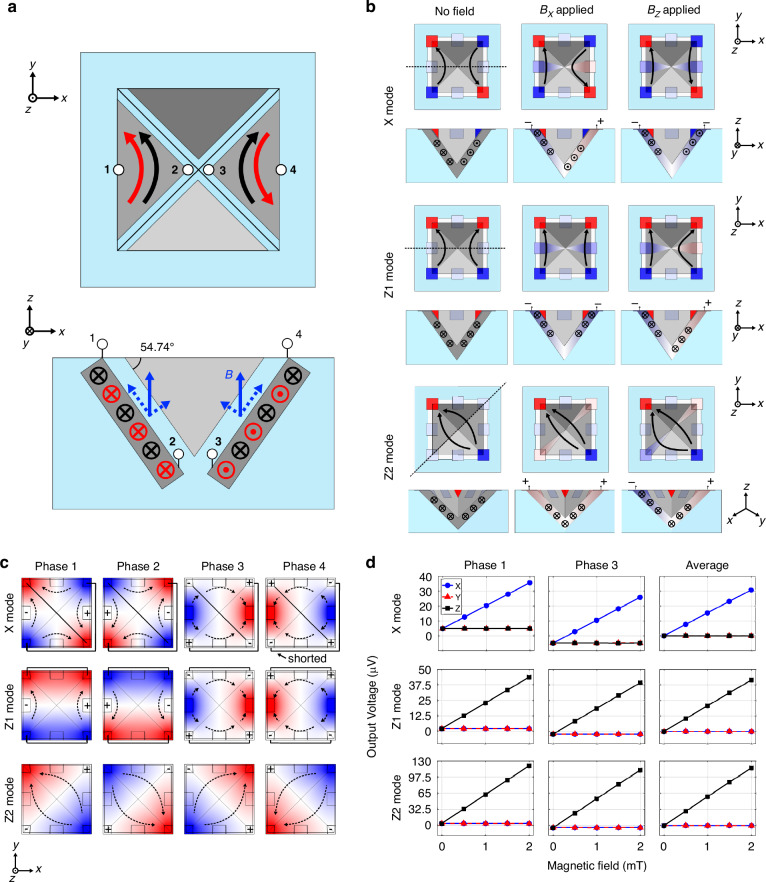
Sensing mechanism of the 3-axis pyramid device. **a** Simplified model of the pyramid device. The sensor is treated as four separated Hall plates. The black arrows represent the biasing configuration for *z*-field detection, while the red arrows display the biasing scheme for *x*-field sensing. **b** Three sensing modes: X, Z1, and Z2. The black arrows represent the carrier velocities, and their deflection displays qualitatively the effect of an applied magnetic field. **c** The spinning sequence of modes X, Z1, and Z2. In mode X and Z1, the opposite and adjacent corner contacts are respectively shorted. The black arrows represent again the electrons’ velocities. **d** COMSOL simulations of the spinning sequences. Two orthogonal phases are simulated and averaged per mode, to reproduce current-spinning. Both sensitivity and crosstalk are reported

In this analysis, let us consider the two devices that are symmetric through the *yz* plane, as depicted in Fig. 2a. To begin, let us push two parallel and identical currents in the Hall sensors. Under the listed assumptions, the Hall voltage produced by a single element between its middle side contact (1) and the apex of the pyramid (2) in the presence of an arbitrary magnetic field can be written as:

1 $${V}_{\text{H}}^{12}={S}\sin (\theta ){B}_{x}+{S}\cos (\theta ){\text{B}}_{\text{z}}$$

where $\theta =\,54.74^{\circ}$ is the etching angle and *S*, instead, is the sensitivity of a standard Hall plate:

2 $$S=\frac{1}{2}{G}_{\text{H}}\frac{{\text{r}}_{\text{H}}}{{ned}}{I}_{\text{supply}}={\mu }_{\text{H}}\frac{{G}_{\text{H}}}{{(L/W)}_{\text{eff}}}{V}_{\text{supply}}$$

In the equation, *G*_H_ is the geometric correction factor, r_H_ is the Hall factor, *n* is the concentration of carriers, *e* is the elementary charge, *d* is the thickness of the plate, ${\mu }_{\text{H}}={\text{r}}_{\text{H}}$ *μ* is the Hall mobility and (*L/W*)_eff_ is the effective number of squares. The extra 1/2 factor, not present in a typical Hall plate, is due to the fact the total supply current *I*_supply_ is split between two elements.

If we consider the opposite device, the Hall voltage measured between the apex of the pyramid (3) and its middle side contact (4) can instead be written as:

3 $${V}_{\text{H}}^{34}=-S^{\prime}\; {\mathrm{sin}}(\theta ){B}_{x}+S^{\prime}\; {\mathrm{cos}}(\theta ){B}_{z}$$

Equations 1 and 3 are a set of linear equations from which the *x*- and *z*- components of the field can be extracted. The two equations can be added together to obtain:

4 $${V}_{{\text{H}}}^{z}={V}_{{\text{H}}}^{12}+{V}_{{\text{H}}}^{34}=(S+S^{\prime})\cos (\theta ){B}_{z}+(S-S^{\prime})\sin (\theta ){B}_{x}$$

However, *S* = *S’* because the two devices are identical. As a consequence, the equation becomes:

5 $${V}_{{\text{H}}}^{z}=2{S}\cos (\theta ){B}_{z}$$

The Hall voltage ${V}_{{\text{H}}}^{z}$ is, therefore, only sensitive to the out-of-plane component of the field, and the crosstalk to the in-plane component is discarded by symmetry. To obtain the in-plane sensitivity we can subtract Eqs. 1 and 3, or reverse the direction of the current that flows in the left device. In this way, the measured Hall voltage can be written as:

6 $${{\text{V}}}_{{\text{H}}}^{x}={V}_{{\text{H}}}^{12}-{V}_{{\text{H}}}^{34}=2{S}\sin (\theta ){B}_{x}$$

If the two sloped devices are slightly different due to some fabrication inaccuracies or asymmetries, *S* is not equal to $S^{\prime}$ anymore and some crosstalk arises. In a more general situation where each device might additionally present some imperfections, we may write:

7 $$\left[\begin{array}{c}{V}_{{\text{H}}}^{x}\\ {V}_{{\text{H}}}^{y}\\ {V}_{{\text{H}}}^{z}\end{array}\right]=\left[\begin{array}{cc}\begin{array}{c}{S^{\prime\prime}}_{{xx}}\\ {S^{\prime\prime}}_{{yx}}\end{array} & \begin{array}{c}\begin{array}{cc}{S^{\prime\prime}}_{{xy}} & {S^{\prime\prime}}_{{xz}}\end{array}\\ \begin{array}{cc}{S^{\prime\prime}}_{{yy}} & {S^{\prime\prime}}_{{yz}}\end{array}\end{array}\\ {S^{\prime\prime}}_{{zx}} & \begin{array}{cc}{S^{\prime\prime}}_{{zy}} & {S^{\prime\prime}}_{{zz}}\end{array}\end{array}\right]\left[\begin{array}{c}{B}_{x}\\ {B}_{y}\\ {B}_{z}\end{array}\right]$$

where the ${S^{\prime\prime}}_{\text{nm}}$ terms belong to the sensitivity matrix that relate the Hall voltage components to the three components of the magnetic field. The first-principles analytical determination of the $S^{\prime\prime}$ coefficients is out of the scope of this work. ${\text{V}}_{\text{H}}^{y}$ has been reported for the sake of completeness, despite the fact that the *y*-fields can only be detected by the remaining sloped faces of the pyramid device.

Comparing Eq. 7 with Eq. 4, it can be noticed that an additional crosstalk component is present in ${\text{V}}_{\text{H}}^{\text{x}}$ from the other lateral dimension (*y*). The crosstalk to the *y*-field is due to inaccuracies within the same device, which slightly tilt the currents. Since these currents do not flow anymore exclusively along the *y*-axis, a small Hall voltage is produced by the *y*-component of the magnetic field.

Lastly, a practical pyramid device is more complex since the current also flows across the device edges, which were previously assumed isolated. Even for a perfectly symmetric and uniform sensor, the sensitivity *S* of a single sloped face presents *G*_H_ and (*L/W*)_eff_ factors that differ from the isolated assumption. In the pyramid device, in fact, the current can split between adjacent faces and re-enter the same element at a different location. This implies that different modes might display different geometry responses. What is more, now the apex contact is the same (points (2) and (3) in Fig. 2a), and due to superposition we can simply measure the total voltage between the two sidewall contacts (points (1) and (4)). Therefore, the apex does not require an external electrical contact.

### Operation modes

For this study, we present three different operation modes named X/Y, Z1, and Z2, represented in Fig. 2b. In the X/Y mode, an alternating biasing pattern is applied, resulting in the flow of four anti-parallel currents. Two middle-side contacts, aligned along the *x*-axis, detect the Hall voltage variation due to an *x*-field. The other two middle-side contacts, instead, detect the *y*-field. The two antiparallel currents respond to the in-plane field with equal but opposite Lorentz forces. The electrons that flow in the first sloped side move closer to the sensing contact, while the electrons in the second face are dragged towards the apex of the pyramid. In the presence of a *z*-field, the electrons on the two faces are dragged by the same Lorentz force in the same direction, and the crosstalk is removed by symmetry. In the Z1 mode, the bias pattern is symmetric with respect to the *yz* plane. Two parallel currents flow in the devices along the *y*-direction. The working principle is the same as in the X/Y mode, but the sensitivity and crosstalk components are exchanged. The Z2 mode presents only one pair of supply contacts, located at opposite corners. The Hall voltage is sensed at the remaining corner contacts. Once the supply is applied, the current splits into two branches that flow parallel to each other, similar to the Z1 mode.

### Current-spinning schemes

In a real device, the output voltage (*V*_out_) can be written as:

8 $${V}_{{\text{out}}}={V}_{{\text{H}}}+{V}_{{\text{off}}}+{v}_{{\text{n}}}(t)$$

where *V*_H_ is the Hall voltage, *V*_off_ is the offset voltage and *v*_n_ is the noise of the system. The offset represents the output signal at zero applied magnetic field and arises due to asymmetries provoked by mismatches, lithographic inaccuracies, and doping inhomogeneities. Typically, the offset is similar or even higher than the signal itself. Various methods have been developed to mitigate the offset, and the most commonly adopted is a dynamic offset cancellation technique known as current-spinning. Current-spinning involves continuously switching the supply and sensing contacts over time. This process modulates the offset at higher frequencies while keeping the Hall voltage constant^16^. Despite this technique, some offset remains, known as the residual offset. In typical Hall devices, the offset reduction achieved is 500–1000 times the initial raw offset^17^.

The pyramid device, which features eight contacts, can be read out by a substantial number of biasing and sensing configurations. The objective is to identify orthogonal phases among these configurations to effectively cancel out the offset and inverse polarity phases to counteract thermal gradients. Moreover, these configurations must be sensitive only to the specific component of the magnetic field we aim to detect, with minimal crosstalk. For each mode proposed in the previous paragraph, the corresponding spinning sequence was identified and they are illustrated in Fig. 2c. The first sequence represented is the X mode spinning scheme. The Y mode is not shown since it is identical to the X mode, but 90° rotated. Opposite corner contacts must be shorted to properly spin the device, either by a metal line or a switch. The reason for this short is to reduce the total number of electrical connections to six. However, since we use only four electrical connections for either X or Y detection, it can be considered a four-contact device, similar to a standard PHD. This simplification makes the spinning sequence straightforward. Notably, phases 1 and 2, and phases 3 and 4 are inverse, achieved by reversing the sign of the supply and sense contacts. Phases 1 and 3, as well as phases 2 and 4, are orthogonal and obtained by swapping the supply and sense terminals. For the Z1 spinning sequence, the approach is similar to mode X/Y, but in this case, adjacent pairs of corner electrodes are shorted. Both modes X/Y and Z1 exhibit two orthogonal phases that are not symmetric since supply and sense contacts are of two different types, i.e., either corners or middle-side terminals. Lastly, the Z2 spinning sequence does not require any short, it uses only the four corner contacts and the spinning sequence is exactly the same as that of a standard PHD^18^. To prove the concept and functionality of each spinning sequence, two orthogonal phases were simulated using the finite element method (Fig. 2d). The simulation results indicate that, for a symmetric device, each phase of each mode does not exhibit crosstalk. Additionally, the offset related to the mesh is perfectly cancelled by averaging the results of phases 1 and 3, since the simulation model assumes perfect electrical linearity.

## Results

### Sensitivity and crosstalk

**Fig. 3:**
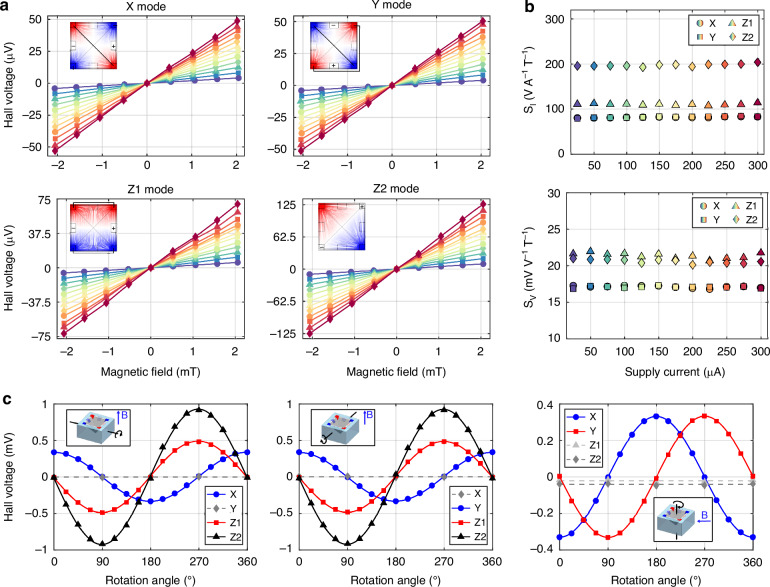
Sensitivity and crosstalk measurements. **a** Hall voltage in function of the magnetic field, for twelve equidistant supply currents in the range 25–300 μA, measured for sensor P1. **b** Current- (*S*_I_) and voltage-(*S*_V_) related sensitivities of sensor P1, for twelve different supply currents. **c** Hall voltage in function of the rotation angle, for a 100 μA supply current and a magnetic field of 48 mT, measured for sensor P4. The solid line is the fit employed to extract the crosstalk. The rotation angle and direction of the magnetic field are reported in the inset of each graph

To evaluate the performance of each detection mode, the sensitivity to magnetic field was extracted when supplied with twelve equidistant supply currents in the range 25–300 μA. Figure 3a displays the Hall voltage for the X, Y, Z1, and Z2 modes. As expected, the response is linear. The output voltage is in the microvolt order, as typical for Hall devices in the milliTesla range. In general, the sensitivity of Hall-effect devices is reported normalized to the supply current or supply voltage. Two metrics known as current-related sensitivity (*S*_I_) and voltage-related sensitivity (*S*_V_), defined as the sensitivity normalized by the supply current or voltage, are reported in Fig. 3b in function of the supply current. Considerable fluctuations, on the scale of 1 to 5%, are present because of the measurement noise. Therefore, related second-order effects such as heating and junction field effect are not resolvable in this supply range.

The sensitivity measurements were repeated for three different samples: P1, P2, and P3. The *S*_I_ and *S*_V_, as well as the input resistances measured at 100 μA for the three samples, are reported in Table 1. The difference in sensitivity and resistance is arguably due to process spread, which we assume is caused by the non-uniform distribution of the spray-coated photoresist. In fact, the inhomogeneity of the process likely resulted in slightly larger or thinner features depending on the position of the device on the wafer, but this is not quantified further in this work.

**Table 1:** Magnetic field sensitivity, crosstalk, and input resistance for two orthogonal phases of sensors P1 - P4

Sensor	Mode	*S*_I_	*S*_V_	*R* (kΩ)		*C* (V A^−1^ T^−1^)		
		(V A^−^^1^ T^−1^)	(mV V^−1^ T^−1^)	Ph1	Ph3	X	Y	Z
P1	X	81.7	17.1	3.74	5.74	–	–	–
	Y	82.2	17.1	3.74	5.79	–	–	–
	Z1	111	21.4	4.71	5.74	–	–	–
	Z2	198	20.6	9.26	9.60	–	–	–
P2	X	73.7	15.9	3.58	5.58	–	−1.44 ± 0.44	−1.54 ± 0.90
	Y	72.5	15.6	3.60	5.64	0.69 ± 0.50	–	2.59 ± 0.79
	Z1	103	20.0	4.57	5.62	−1.4 ± 1.8	−0.9 ± 1.7	–
	Z2	190	20.0	9.29	9.40	1.9 ± 3.3	−2.5 ± 3.2	–
P3	X	64.1	14.8	3.30	5.23	–	–	–
	Y	67.2	15.7	3.29	5.19	–	–	–
	Z1	94.8	19.6	4.28	5.30	–	–	–
	Z2	178	20.2	8.66	8.67	–	–	–
P4^a^	X	68.6	14.9	3.61	5.67	–	0.38 ± 0.31	0.79 ± 0.87
	Y	68.6	14.8	3.65	5.72	−0.63 ± 0.57	–	1.07 ± 0.72
	Z1	100.3	19.4	4.69	5.65	2.3 ± 1.3	1.68 ± 0.78	–
	Z2	190.4	19.7	9.68	9.64	3.3 ± 2.7	3.8 ± 1.7	–

^a^*S*_I_ extracted from Fig. 3c interpolation

The crosstalk of each mode with the remaining components of the magnetic field was evaluated by rotating P2 and a fourth sample (P4) in the magnetic field. Three rotation axes were employed, which coincide with the *x*-, *y*-, and *z*-axis of the sample coordinate system. The rotation axis was aligned orthogonal to the applied field, and the output voltages of the modes were evaluated for each rotation angle and three magnetic fields: 0, 24, and 48 mT. The Hall voltages for a magnetic field of 48 mT are reported in Fig. 3c. The rotation axes and the direction of the field are displayed in the inset of each graph.

Equation 7 might be used in theory to fit the experimental data. These fitting functions, however, can be simplified by including the constraints introduced by the experimental setup. First of all, since the rotation axis always coincides with the *x*-, *y*- or *z*- sample axis, the corresponding component *B*_*x*_, *B*_*y*_ or *B*_*z*_ is equal to zero. Furthermore, the two remaining components are obtained by projecting the constant magnetic field onto the sample reference system, which means that they vary with the sine and cosine of the rotation angle. Consequently, the simplified fitting functions can be written as:

9 $${V}_{{\mathrm{H}},{\mathrm{m}}}=({S}_{{\mathrm{I}}}\sin (\phi +\psi )+C\cos (\phi +\psi )){I}_{{\mathrm{supply}}}B$$

10 $${V}_{{\text{H}},{\text{n}}}=(C\sin (\phi +\psi )+{S}_{{\text{I}}}\cos (\phi +\psi )){I}_{{\text{supply}}}B$$

where *ϕ* is the rotation angle, *ψ* is the constant phase shift (for temporal alignment), and *S*_I_ and *C* are the fitted parameters corresponding to current-related sensitivity and current-related crosstalk. For each graph of Fig. 3c, Eq. 9 is used to fit one component (*m*), while Eq. 10 is used to fit the other (*n*). The fitted *C* values for each orientation are reported in Table 1. This crosstalk varies from mode to mode and with the magnetic field component, but it is below 4.7% (< 5.7 V A^−1^ T^−1^). The best value extracted is 0.55% (−0.38 V A^−1^ T^−1^), and it was achieved with mode X, sample P4 and *y*-magnetic field applied.

### Raw and residual offset

**Fig. 4:**
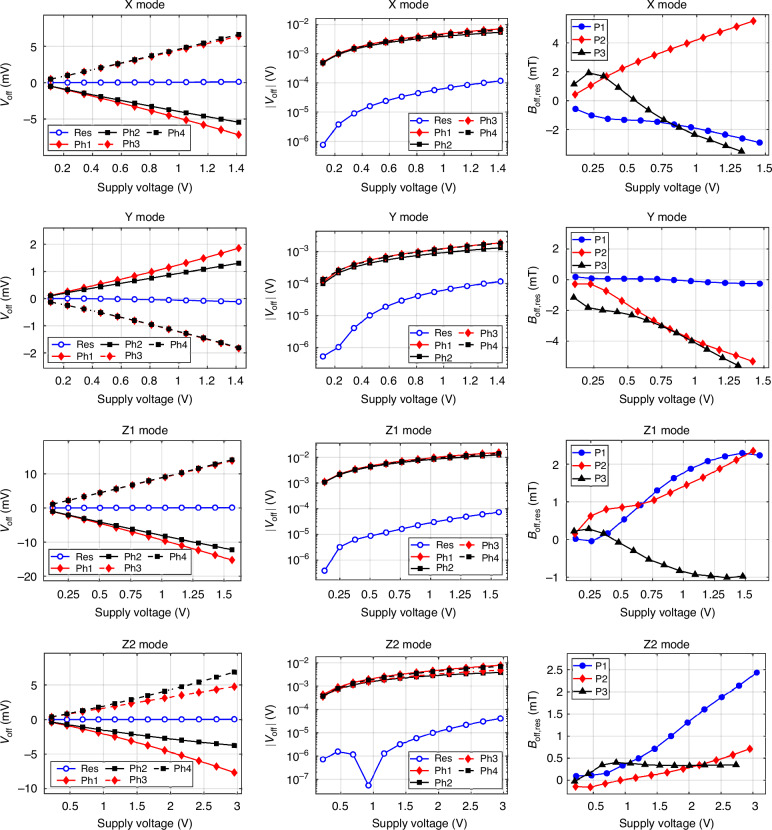
Raw and residual offsets of the pyramid device. The raw offset voltages (*V*_off_) of device P2 are reported in both linear and semilogarithmic scales, together with the residual offset. The equivalent magnetic residual offsets (*B*_off,res_) for the three sensors characterized are reported in linear scale

As mentioned in “Current-spinning schemes”, the tested devices display an offset voltage that can be effectively reduced by means of current-spinning. To validate the spinning configurations, the output voltage is measured and averaged over the four phases of each mode’s spinning sequence. In reality, the full spinning cycles include four additional phases to eliminate the offset of the multimeter, obtained by switching the sense contact polarity in each of the unique four phases. This adds redundancy to the spinning sequence which can be improved in microsystem implementation^19^. The measurement is performed for twelve equidistant currents in the range of 25–300 μA and repeated for devices P1, P2, and P3. Figure 4 depicts the raw equivalent offset of the four phases, as well as the residual offset, on linear and logarithmic scales for modes X, Y, Z1, and Z2. The raw offsets exhibit slight non-linear trends, which can be associated with second-order effects such as Seebeck voltage due to internal thermal distributions, the junction field effect, and packaging stresses^20,21^. The four-phase spinning sequence effectively removes external thermal gradients and geometric offsets but is unable to remove the second-order terms, causing the residual offset. The residual offset achieved is stochastic and depends on the sample, mode, and supply voltage. For all characterized samples and modes, the offset reduction factor is between one and three orders of magnitude.

To coherently compare different devices and technologies, the residual offset is typically scaled by the sensitivity and reported in magnetic field units. This quantity, referred to as the equivalent magnetic offset, sets a bound on the device’s limit of (zero magnetic field) detection. The equivalent residual offsets for all devices and modes are also represented in Fig. 4. No common or clear trend can be observed within the different samples for the same mode, apart from a general increase with the supply voltage. In addition, some curves do not even show a monotonic behavior. This suggests that the residual offset is not ruled by a single effect but rather by multiple physical causes that become more or less prominent at higher supply voltages^22^. At 0.5 V, the general voltage supply at which VHDs are operated, the residual offset varies between 0.1 mT and 2.5 mT.

### Noise

The final figure of merit evaluated for the novel Hall-effect device is its noise performance. Alongside the offset, the noise defines the sensor’s detection limit, but also its resolution. The noise spectral density was evaluated for modes X, Z1, and Z2, and at three different supply voltages: 1.31 V, 0.654 V, and 0.331 V. Mode Y is not reported since it is nominally identical to mode X. An additional measurement was taken with no supply voltage applied, with the input terminals of the pyramid device left floating. The frequency range for these measurements spanned from 10 Hz to 100 kHz.

**Fig. 5:**
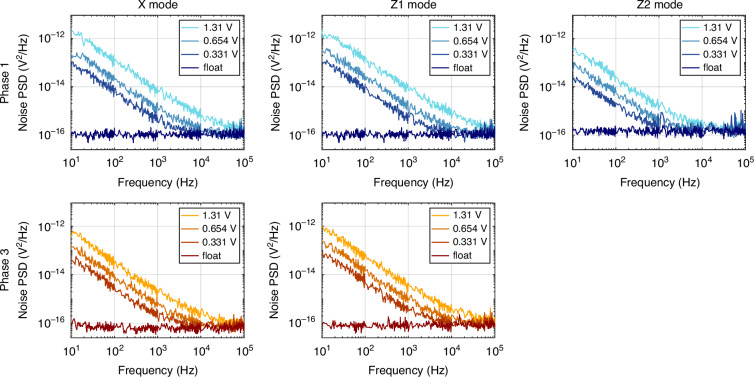
Noise power spectral densities (PSD) of sensor P1. The noise is measured for three supply voltages at room temperature, in two orthogonal phases, for mode X, Z1, and Z2

For modes X and Z1, the noise spectral density was measured for two orthogonal phases, i.e., phase 1 and phase 3. Unlike Z2, modes X and Z1 exhibit asymmetric orthogonal phases, leading to distinct power density spectra that are intrinsically different. Figure 5 displays the characterization results.

As with many semiconductor devices, the sensor displays two primary noise components: flicker noise and thermal noise. Flicker noise can be described by the Hooge model^23^, following the equation:

11 $${S}_{{\text{vf}}}^{2}={V}_{{\text{supply}}}^{2}\,\frac{{\alpha }_{{\text{H}}}}{N}\,\frac{1}{{f}^{\gamma }}$$

where ${S}_{\text{vf}}^{2}$ is the power spectral density of the flicker noise, *V*_supply_ is the supply voltage, α_H_ is the Hooge parameter, *N* is the total number of carriers, and *γ* is a dimensionless factor that should be close to 1. The intersection frequency between the extrapolated flicker noise and the thermal noise floor is known as the corner frequency. It has been demonstrated that current-spinning modulates flicker noise if the spinning frequency exceeds the corner frequency. *γ* and α_H_/*N* were extracted for a supply voltage of 1.31 V by fitting the 1/*f* portion of the spectrum with the Hooge model. In addition, the corner frequencies and thermal floors were derived. These parameters are reported in Table 2. For all modes and phases, the *γ* factor is higher than 1, which implies the presence of traps with a nonuniform energy distribution^24^. The thermal noise differs between modes and phases, as expected with the varying input impedances reported in Table 1, and it is around $10\text{nV}/\sqrt{\text{Hz}}$. This corresponds to an equivalent magnetic noise level of around $0.5\mu \text{T}/\sqrt{\text{Hz}}$ at 1.31 V. The corner frequencies are below 40 kHz, for a supply voltage of 1.31 V.

**Table 2:** Power spectra fitted parameters at 1.31 V, extracted from P1

Mode	Phase	α_H_/*N*	*γ*	Thermal floor	Corner frequency
				(nV/$$\sqrt{\rm{Hz}}$$)	(kHz)
X	Phase 1	2.29 × 10^−^^11^	1.25	10.4	27.4
	Phase 3	5.55 × 10^−^^12^	1.23	8.07	16.0
Z1	Phase 1	2.14 × 10^−^^11^	1.21	10.4	38.0
	Phase 3	9.26 × 10^−^^12^	1.22	9.02	22.1
Z2	Phase 1	4.17 × 10^−12^	1.22	11.9	6.95

## Discussion

Based on the results obtained, we state that the device is a valid and promising solution for 3D magnetic field sensing. The proposed model highlights important similarities with PHDs and provides a starting point for the sensor optimization. For instance, Eq. 2 highlights how low doping is beneficial for both *S*_I_ and *S*_V_. In addition, it implies that the *S*_I_ is enhanced by shallow diffusions. This is an evident advantage over VHDs, which require technologically-challenging deep and narrow diffusions to achieve a similar enhancement. On the other hand, the device relied on spray-coating for its manufacturing, which is not commonly employed in CMOS technology. Further technological development is therefore necessary to achieve smooth CMOS integration.

A summary of the 3-axis Hall pyramid device performances, together with the existing state-of-the-art 3-axis sensors and 5-contact vertical Hall devices (5CVHD), is reported in Table 3. The device exhibits high *S*_I_, higher than several state-of-the-art devices such as the PHD + VHDs configuration and the hexagonal Hall device, but relatively low *S*_V_. This can be attributed to the high doping of the device, approximately 5 × 10^16^ cm^−3^, which degrades mobility and, thus, sensitivity. What is more, *S*_V_ might be increased by improving the geometry of the device^15^. The best *S*_I_ and *S*_V_ ratios between in-plane and out-of-plane modes are 0.74 and 0.83. Although the device is not optimized, it exhibits high isotropy which is already better than many existing 3-axis Hall sensors, such as the PHD + VHDs configuration and the IMC Hall. This can be further improved by tuning the sensor geometrical parameters.

**Table 3:** Comparison of the novel 3-axis pyramid device with the state-of-the-art

Sensor	*S*_I_ (V A^−1^ T^−^^1^)		*S*_V_ (mV V^−^^1^ T^−^^1^)		*B*_off,res_ (1 V)	Spatial resolution
	XY	Z	XY	Z	(mT)	(μm)
Pyramid	64.1–82.2	94.8–198	14.8–17.1	19.6–21.4	0.2–4	50
5CVHD^17^	40.5	–	17.2	–	0.7–3.4	–
5CVHD^25^	46.4	–	32	–	1–4	–
5CVHD^8^	~120	–	35.1	–	0.6	–
PHD + VHDs^6,11^	6.27	90.1	1.9	27.3	–	44
3-axis planar^9^	46–827	17–909	20–54	20–33	–	50–500
Hexagonal^10^	8.6–8.8	8.7	33.0–33.9	33.3	0.04	850
IMC Hall^13^	~1500	250	~300	50	~0.03^a^	2000

^a^Estimated from the data reported in the paper^13^

The crosstalks exhibited by the devices are reasonably low (< 4.7%). As explained in “Current-spinning schemes”, their origin is due to asymmetries in the structure. Despite sharing the same fundamental causes with the offset, crosstalk cannot be current-spun. The only method to reduce this effect is to improve the manufacturing process, minimizing mismatches and inhomogeneities.

These first-generation sensors, however, display very high offsets compared to PHDs. The raw offset is around 50 mT to 100 mT, which suggests strong underlying asymmetries related to the patterning and implantation of tilted (111) silicon planes. The residual offset (*B*_off,res_) after current-spinning is reduced to the millitesla order, which is similar to 5CVHDs^8,17,25^. It is interesting to notice that modes X,Y, and Z1 present raw offsets that in phases 3 and 4 almost overlap, while in phases 1 and 2 they diverge already at low supply voltages. This is similar to the behavior exhibited by VHDs^26^. On the other hand, mode Z2 residual offset has more similarities with PHDs, almost one order of magnitude better than the other three modes, but still much higher than PHDs^27^. This hints at the presence of some extra components of residual offset beyond typical Hall plates. The residual offset curves do not display a clear trend, so multiple sources are involved. For instance, some samples display a quadratic offset voltage increase (i.e., a linear equivalent offset increase) for high supply, which suggests Joule heating and strong temperature gradients. Other samples, instead, increase and then steadily decrease, hinting at the competing mechanisms. The resistance non-linearity of < 4.7% V^−^^1^ at 0.5 V implies that the junction field effect is present and plays a role, but it is less prominent than for VHDs with non-linearities around 10% V^−1^ ^8^.

Finally, it is also worth noting that the device exhibits relatively lower active volume and footprint than PHDs and other state-of-the-art devices. Smaller devices tend to display larger raw offsets produced by lithographic inaccuracies, but also higher residual offsets due to velocity saturation caused by the higher electric fields^8^. Therefore, the results obtained with large footprint devices, such as the hexagonal Hall sensor^11^, and the pyramid device may not be directly comparable. A similar argument can be made for flicker noise, which scales down with the sensor’s active volume since the 1/*f* noise depends on the total number of dopants (Eq. 11).

The high flicker noise is not a significant problem since it can be modulated by the spinning sequence as long as the spinning frequency is higher than the corner frequency^28^. The corner frequencies, 6.95 kHz to 38.0 kHz at 1.31 V, are sufficiently low to properly spin the devices with interfacing electronics. However, the origin of the high flicker noise could highlight further problems and/or properties of the device. For instance, one of the possible causes of offset and flicker noise is mobile charges in the passivation layers, which move around in the presence of an electric field and behave as trap levels^29^. This is consistent with measured *γ* > 1, as well as with the high α_H_/*N*. Other causes might be trap levels related to the quality of the (111) etched planes, asymmetric local heatings and noisy contacts^30^.

In the current state, the industrial applicability of the device is limited to higher magnetic field applications such as high-power 3D current sensing. The sensitivity of the device is, in fact, comparable to the state-of-the-art VHDs, but the residual offset is in the millitesla range. To enable further applications such as angular and position sensing, the residual offset should be reduced by at least one order of magnitude. For example, to achieve an angular error of 0.2° with a 100 mT magnet, an offset below 0.2 mT is required^25^. This goal can be achieved by solving some of the discussed problems such as asymmetries, heating effects and mobile charges by improving the sensor design and fabrication. In addition, more operation modes could be investigated and tested, which might achieve better performance.

## Materials and methods

### Fabrication and process flow

**Fig. 6:**
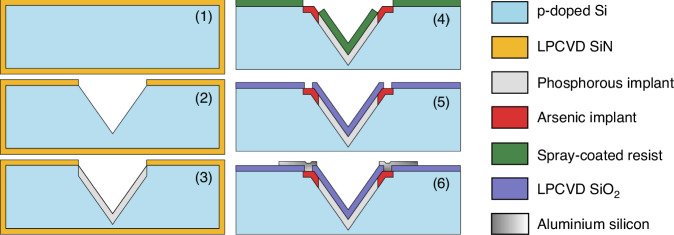
Pyramid sensor process flow. (1) LPCVD SiN deposition. (2) Pyramid cavity etching. (3) Active area implantation (4) n+ contact implantation (5) Device passivation (6) AlSi sputtering and patterning

The pyramid sensors were realized with a process flow that leveraged CMOS technology and standard MEMS micromachining (Fig. 6). First, the p-doped (100) silicon wafer (resistivity from 2 Ω·cm to 5 Ω·cm) was covered with low-pressure chemical vapor deposited (LPCVD) silicon nitride (SiH_2_Cl_2_ 340 sccm; NH_3_ 60 sccm; 850 °C). The nitride layer was patterned by reactive ion etching (RIE) using C_2_F_6_, followed by the anisotropic wet etching of the silicon with tetramethylammonium hydroxide (TMAH) 25% at 90 °C. A 20 nm silicon oxide dirt barrier was then grown through dry oxidation at 950 °C. After that, the pyramidal cavities were ion-implanted with phosphorus (energy: 100 keV; dose: 4.5 × 10^12^ cm^−2^, tilt: 0°), employing again the silicon nitride layer as a self-aligned implantation mask. The nitride was removed with phosphoric acid at 155 °C, and the devices were annealed at 1150 °C for 60 min to activate and diffuse the dopants. The goal net doping and junction depth were 5 × 10^16^ cm^−3^ and 1 μm. After the growth of a second 20 nm dirt barrier, the sloped sidewalls were conformally covered with spray-coated photoresist and patterned to define the regions to be implanted with arsenic (energy: 40 keV; dose: 5 × 10^15^ cm^−^^2^, tilt: 0°). The photoresist was then removed, and 300 nm of LPCVD silicon oxide were deposited (TEOS bubbler 50 °C; 250 mTorr; 700 °C) to play the role of passivation layer. The devices were annealed at 1000 °C for 30 min. The oxide was patterned and etched with buffered hydrofluoric acid (BHF) 1:7 diluted with water to open the vias, and 500 nm of aluminum-silicon (1%) was sputtered onto the wafer at 350 °C. The aluminum-silicon was wet-etched with PES (HNO_3_, H_3_PO_4_, acetic acid and water) to define the pads and the metal interconnections, and subjected to a polysilicon dip-etch with HNO_3_ and HF to remove the polysilicon grains. Lastly, the metal lines were annealed at 400 °C in a reducing atmosphere (N_2_ 3.0 L/min, H_2_ 0.2 L/min). In the back end of line, the wafers were diced and the devices were glued and wirebonded to a carrier PCB.

### Experimental setups and procedures

Four experimental setups were employed in this work to measure sensitivity, crosstalk, offset, and noise spectrum.

For the sensitivity characterization, a Helmholtz coil was employed to apply a uniform magnetic field in the range 0–2 mT. The coil was driven by a TENMA 72-2645 programmable power source. The sensor was supplied with current by a Keithley 2400 sourcemeter, and the output voltage was measured by an Agilent 34401A multimeter. The spinning sequence was actuated by an Agilent U2751A switch matrix. The measurements were repeated for twelve equidistant currents in the range 25–300 μA, and averaged over 20 eight-phases spinning cycles. To perform the measurements for negative magnetic field the coil power supply terminals were swapped. The offset measured during the sweeps is then subtracted from the output voltage, and the curves are interpolated with a linear regression to obtain the sensitivity. The supply voltage was averaged in the *S*_V_ calculation.

For the crosstalk measurements, another Helmholtz coil with a maximum field of 48 mT was employed. The sample was placed inside the coil and manually rotated through a 3D printed knob, with a graded scale with a rotation error of up to 3°, connected to the sample holder. The sample was rotated over 17 equidistant angles to perform a complete 360° rotation. For each rotation angle, the device was supplied with a 100 μA current. The device was current-spun in each measurement and averaged over 2 to 21 cycles. The output voltage was measured for fields of ambient field (~0 mT), 24 mT and 48 mT, and the slope and intercept of the output voltage in function of the field were extracted. To remove eventual magnetization effects, external constant magnetic fields, or drifts, the offset was subtracted from the output voltage. The Hall signal in function of the rotation angle was then interpolated with Eq. 9 or Eq. 10 to obtain the sensitivity and crosstalk.

For the offset measurement, the sample was placed in a zero-Gauss chamber to shield it from external magnetic fields. The current supply was swept from 25 μA to 300 μA with step of 25 μA. For each current, every phase and current-spun output was averaged over 50 cycles. The equivalent magnetic residual offset was obtained by dividing the residual offset by the sensitivity obtained from the Helmholtz coil measurements. The sensitivities used are the ones reported in Table 1. The voltage reported on the *x*-axis of the graphs is the average supply voltage of the eight phases.

For the noise measurements, a HP4395A spectrum analyzer was used to extract the power spectral density. The device was supplied with 1.3 V rechargeable battery. A low-pass filtered voltage divider was adopted to split the voltage by half or one-fourth. The output voltage was then amplified by SR560 low-noise voltage amplifier. 50 Hz and integer multiples were skipped to avoid peaks from the power grid. The sample and the biasing circuit were stored, during the measurement, in a zero-Gauss chamber. The 1.31 V biases curves were fitted with the Hooge model to obtain the parameters reported in Table 2.

## Conclusion

In this work, we developed a novel type of 3-axis magnetic sensor based on the Hall-effect and an inverted pyramidal geometry. The device was realized by combining MEMS micromachining and standard CMOS technology, and it exhibited high in-plane and out-of-plane current sensitivities in the range 64.1–198 V A^−^^1^ T^−1^. The sensor presented low crosstalk, not higher than 4.7%. In addition, we proved that the device can be current-spun, achieving a reduction in raw offset of one to three orders of magnitude. The noise spectral density of the device was measured, and it exhibited corner frequencies lower than 40 kHz at 1.31 V, which means that the device can be spun with modern CMOS technology nodes without introducing extra errors due to the spinning circuitry. Despite the substantial reduction due to current-spinning, the residual offset is still in the millitesla order, which is higher than standard Hall plates but comparable to VHDs. Significant improvement in the design and process flow is possible, but nevertheless the device is a promising solution and a simple alternative to state-of-the-art 3-axis Hall-effect magnetic sensors. The presented device, with proper development, will enable accurate and cost-effective position sensing and angle measurements for automotive, industrial and consumer electronics applications.

## References

[1] Dìaz-Michelena, M. Small magnetic sensors for space applications. *Sensors* **9**, 2271–2288 (2009). 22574012 10.3390/s90402271 PMC3348798

[2] Lenz, J. & Edelstein, S. Magnetic sensors and their applications. *IEEE Sens. J.* **6**, 631–649 (2006).

[3] Elzwawy, A., Rasly, M., Morsy, M., Piskin, H. & Volmer, M. *Magnetic Sensors: Principles, Methodologies, and Applications*, 1–38 (Springer Nature Switzerland, Cham, 2024).

[4] Ripka, P. *Magnetic Sensors and Magnetometers*, Second Edition (Artech, 2021).

[5] Khan, M. A., Sun, J., Li, B., Przybysz, A. & Kosel, J. Magnetic sensors – a review and recent technologies. *Eng. Res. Express* **3**, 022005 (2021).

[6] Pascal, J., Hebrard, L., Frick, V. & Blonde, J.-P. 3D Hall probe integrated in 0.35 μm CMOS technology for magnetic field pulses measurements. In *2008 Joint 6th International IEEE Northeast Workshop on Circuits and Systems and TAISA Conference*, 97–100 (IEEE, 2008).

[7] Popovic, R. The vertical Hall-effect device. *IEEE Electron Device Lett.* **5**, 357–358 (1984).

[8] Ausserlechner, U. Hall effect devices with three terminals: their magnetic sensitivity and offset cancellation scheme. *J. Sens.* **2016**, 5625607 (2016).

[9] Schott, C., Waser, J.-M. & Popovic, R. Single-chip 3D silicon Hall sensor. *Sens. Actuators A:Phys.* **82**, 167–173 (2000).

[10] Sander, C., Leube, C., Aftab, T., Ruther, P. & Paul, O. Isotropic 3D silicon Hall sensor. In *2015 28th IEEE International Conference on Micro Electro Mechanical Systems (MEMS)*, 893–896 (IEEE, 2015).

[11] Sander, C., Leube, C., Aftab, T., Ruther, P. & Paul, O. Monolithic isotropic 3D silicon Hall sensor. *Sens. Actuators A: Phys.* **247**, 587–597 (2016).

[12] Popovic, R. S., Drljaca, P. M., Schott, C. & Racz, R. Integrated Hall sensor/flux concentrator microsystems. In *INFORMACIJE MIDEM-LJUBLJANA*, 215–219 (MIDEM; 1999, 2001).

[13] Schott, C., Racz, R., Manco, A. & Simonne, N. CMOS single-chip electronic compass with micro-controller. *IEEE J. Solid-State Circuits* **42**, 2923–2933 (2007).

[14] Fan, C., Jin, Z. & Chen, J. Current state of triaxial magnetoresistance sensors and their applications: a review. *Sens. Actuators A: Phys.* **377**, 115724 (2024).

[15] Ruggeri, J., Strube, J. & Dowling, K. M. 3D Hall-effect magnetometer using a single inverted pyramid structure. In *2024 IEEE 37th International Conference on Micro Electro Mechanical Systems (MEMS)*, 48–51 (IEEE, 2024).

[16] Munter, P. A low-offset spinning-current Hall plate. *Sens. Actuators A: Phys.* **22**, 743–746 (1990).

[17] Sander, C., Vecchi, M.-C., Cornils, M. & Paul, O. From three-contact vertical Hall elements to symmetrized vertical Hall sensors with low offset. *Sens. Actuators A: Phys.* **240**, 92–102 (2016).

[18] Dowling, K. M. et al. Micro-tesla offset in thermally stable AlGaN/GaN 2DEG Hall plates using current spinning. *IEEE Sens. Lett.* **3**, 1–4 (2019).

[19] Mosser, V., Matringe, N. & Haddab, Y. A spinning current circuit for Hall measurements down to the nanotesla range. *IEEE Trans. Instrum. Meas.* **66**, 637–650 (2017).

[20] Dowling, K. et al. The effect of bias conditions on AlGaN/GaN 2DEG Hall plates. In *2018 Solid-State, Actuators, and Microsystems Workshop*, 194–197 (The Transducer Research Foundation (TRF), 2018).

[21] Ausserlechner, U. Limits of offset cancellation by the principle of spinning current Hall probe. In *IEEE SENSORS*, Vol. **3**, 1117–1120 (IEEE, 2004).

[22] Bellekom, A. A. *Origins of Offset in Conventional and Spinning-Current Hall Plates*. Ph.D. thesis, Delft University of Technology (1998).

[23] Hooge, F. N., Kleinpenning, T. G. M. & Vandamme, L. K. J. Experimental studies on 1/f noise. *Rep. Prog. Phys.* **44**, 479 (1981).

[24] Dowling, K. M. et al. Low offset and noise in high biased GaN 2DEG Hall-effect plates investigated with infrared microscopy. *J. Microelectromechanical Syst.* **29**, 669–676 (2020).

[25] Schurig, E., Schott, C., Besse, P.-A., Demierre, M. & Popovic, R. 0.2 mT Residual offset of CMOS integrated vertical Hall sensors. *Sens. Actuators A: Phys.* **110**, 98–104 (2004).

[26] Paul, O., Raz, R. & Kaufmann, T. Analysis of the offset of semiconductor vertical Hall devices. *Sens. Actuators A: Phys.* **174**, 24–32 (2012).

[27] van der Meer, J., Riedijk, F., van Kampen, E., Makinwa, K. & Huijsing, J. A fully integrated CMOS Hall sensor with a 3.65 μT 3σ offset for compass applications. In *ISSCC. 2005 IEEE International Digest of Technical Papers. Solid-State Circuits Conference, 2005*, Vol. **1**, 246–247 (IEEE, 2005).

[28] Jiang, J. & Makinwa, K. A. A. Multipath wide-bandwidth CMOS magnetic sensors. *IEEE J. Solid-State Circuits* **52**, 198–209 (2017).

[29] Popovíc, R. Hall-effect devices. *Sens. Actuators* **17**, 39–53 (1989).

[30] Barone, C. et al. Apparent volume dependence of 1/f noise in thin film structures:role of contacts. *Rev. Sci. Instrum.* **79**, 053908 (2008). 08/30/2024; 01/06/2025. 18513080 10.1063/1.2929830

